# Assessment of nutritional status in the maintenance of haemodialysis patients: a cross-sectional study from Palestine

**DOI:** 10.1186/s12882-019-1288-z

**Published:** 2019-03-15

**Authors:** Ali M. Omari, Leen S. Omari, Hazar H. Dagash, Waleed M. Sweileh, Nehal Natour, Sa’ed H. Zyoud

**Affiliations:** 1 Department of Medicine, College of Medicine and Health Sciences, An-Najah National University, Nablus, 44839 Palestine; 2 Department of Physiology, Pharmacology, and Toxicology, College of Medicine and Health Sciences, An-Najah National University, Nablus, 44839 Palestine; 3 Public Health Department, College of Medicine and Health Sciences, An-Najah National University Hospital, An-Najah National University, Nablus, 44839 Palestine; 4 Poison Control and Drug Information Center (PCDIC), College of Medicine and Health Sciences, An-Najah National University, Nablus, 44839 Palestine; 5 Department of Clinical and Community Pharmacy, College of Medicine and Health Sciences, An-Najah National University, Nablus, 44839 Palestine

**Keywords:** Haemodialysis, Malnutrition-inflammation scale, Palestine

## Abstract

**Background:**

Protein-energy wasting (PEW) is a relatively prevalent problem among adult haemodialysis patients (HDP). PEW is an important determinant of morbidity and mortality in HDP, therefore it is essential for dietitians to accurately assess malnutrition (MN) in these patients. HDP appear to be more susceptible to developing MN; however, this is not well documented. Therefore this study aimed to assess the nutritional status among HDP and to establish the factors associated with MN in these patients.

**Methods:**

A cross-sectional survey was carried out in Nablus, northern West Bank, in the main haemodialysis (HD) centre at the An-Najah National University Hospital. MN was detected using the malnutrition-inflammation scale (MIS), which involved four major elements: the patient’s related medical history, their body mass index, a physical examination and laboratory parameters.

**Results:**

A total of 174 patients (91 male) were included in the study. Patients were aged 57.7 ± 12.8 years, and the median dialysis vintage was 3 years (interquartile range 1–5 years). HDP, especially the elderly (unstandardized coefficient β, 1.728; 95% CI, 0.700 to 2.756; *P* = 0.001), those with multiple comorbid diseases (unstandardized coefficient β, 1.673; 95% CI, 0.556 to 2.789; *P* = 0.004); those taking multiple chronic medications (unstandardized coefficient β, 1.259; 95% CI, 0.197 to 2.321; *P* = 0.020), or those with a long dialysis vintage (unstandardized coefficient β, 1.449; 95% CI, 0.410 to 2.487; *P* = 0.007), were positively associated with the MIS score in a multivariable linear regression model. Furthermore, HDP living with their family were negatively associated with the MIS score (unstandardized coefficient β, − 2.545; 95% CI, − 4.738 to − 0.352; *P* = 0.023).

**Conclusions:**

The MIS score results indicate that MN is prevalent among HDP. These results demonstrate some correlations between nutritional status and patient characteristics (i.e. clinical and sociodemographic factors). Therefore these findings should help to increase the awareness of healthcare providers for interventions to enhance the nutritional status of HDP, especially those who are elderly, have multiple comorbid diseases, have multiple chronic medications, have experienced a long dialysis vintage or who live alone.

**Electronic supplementary material:**

The online version of this article (10.1186/s12882-019-1288-z) contains supplementary material, which is available to authorized users.

## Background

One expanding global public health challenge is chronic kidney disease (CKD) [1, 2], which is manifested by an irreversible deterioration of kidney function that may eventually lead to end-stage renal disease (ESRD) and require renal replacement therapy such as renal transplantation or haemodialysis (HD) [3, 4]. The main aim of HD is to rebalance the intracellular and extracellular fluid volume that is a characteristic function of normal kidneys. This is performed by transporting solutes (i.e. urea) from the blood to the dialysate and by transporting solutes (i.e. bicarbonate) from the dialysate to the blood [5].

In haemodialysis patients (HDP), it is important to perform an early diagnosis of malnutrition (MN) and inflammation, which are represented by protein-energy wasting (PEW) as they are significant predictors of mortality [6–8], using the best clinically available tools to create specific nutritional strategies that can predict outcomes, evaluate therapeutic responses, and avoid severe nutritional deterioration. This adds to the importance and significance of the presence of nutrition specialists in HD centre for early detection of malnutrition and strategies to be implemented to prevent further deterioration [9].

The malnutrition-inflammation scale (MIS) score, established by Kalantar-Zadeh et al., is a quantitative score measuring the nutritional status and its severity [10]. The MIS was found to be superior to conventional predictors such as serum levels of C-reactive protein (CRP) as well as to other scales used to assess malnutrition among HD patients such as subjective global assessment [11–14]. The MIS includes 10 elements, 70% of which are evaluated subjectively and 30% of which are evaluated objectively. Morbidity and mortality were strongly associated with MIS in several studies in CKD nondialysis patients [15] and HDP [10, 16–18]. Therefore, MIS can provide an easy and economic tool for early screening of potential MN in HD centres. One further advantage of MIS is the fact that it can be accomplished by a trained nurse or any member in the health team.

There are dramatic increase in the numbers of ESRD patients was observed over the last few years in the West Bank, Palestine [19]. In 2017, 1216 patients in the West Bank were documented to have ESRD and require HD, compared to 687 patients in 2014 which showed substantial increase in patients requiring hemodialysis [19]. In Palestine, HD centers were run by the Palestinian Ministry of Health which suffers from under-staffing in all fields due to economic conditions [20]. Therefore, we expect that patients undergoing hemodialysis in Palestine may not receive adequate education regarding nutritional behavior or periodic nutritional assessment by experts to avoid any health complications. Numerous studies regarding HDP were conducted and published in Palestine [21–26]; however, no single study emphasized the association and prevalence of MN among HDP. Therefore this study was conducted to determine the relationship between regular HD and the development of MN. The results of this study may help healthcare providers to early implement strategies needed to improve HD outcomes and provide new data about the value of MN in HDP.

## Methods

### Study design

The research approach employed a descriptive cross-sectional design to achieve the study objectives.

### Study setting

This study was carried out in Nablus, northern West Bank, in the main HD department at the An-Najah National University Hospital. HD services offered by the centre extend to a total population of over 300,000 people in the Nablus district.

### Study population, sample size calculation and sampling procedure

According to the 2016 Health Annual Report from the Palestinian Ministry of Health [27], 210 dialysis patients were being treated at the An-Najah National University Hospital served by 33 HD machines. For this study, all patients attending the HD unit and who met our inclusion criteria were approached and asked to participate in the study. During the research period between July and December 2017, 192 HDP were invited to participate in the study.

### Inclusion and exclusion criteria

Patients were included in the study if they were ≥ 18 years old, conscious and alert, receiving HD for ≥6 months, receiving dialysis twice weekly (minimum) with at least 3 h per session and if their medical file contained all the demographic, clinical and biochemical data required for the study. Patients were excluded if they lacked the physical or mental ability to communicate with the interviewer.

### Data collection instrument

A data collection instrument was used that contained three sections (Additional file 1):

- The first section covered social and demographic factors (i.e. age, sex, residency, job, dialysis centre visited, education level, smoking status, marital status and monthly income).
- The second section discussed the clinical status of the patients (i.e. the dialysis vintage in years, the dialysis frequency per week, body mass index (BMI), the number of chronically used medications and the number of chronic diseases).
- *The third section included the* MIS and involved four major parts: the patient’s related medical history, their BMI, a physical examination and laboratory parameters [10]. The patient’s medical history included weight change, gastrointestinal symptoms, dietary food intake, functional capacity and comorbidities (i.e. dialysis duration in years). The physical examination detected subcutaneous fat loss and signs of muscle wasting. Laboratory parameters included serum levels of total iron binding capacity (TIBC) and albumin. The severity of the MIS is composed of four grades for each component which are ranked from zero (normal) to three (very severe). The overall score of the 10 MIS components ranges from 0 to 30, with high scores indicating increased severity [28]. The online calculator, http://www.touchcalc.com/calculators/mis, was used to calculate the MIS score*.* According to a case–control study by Naini et al. [29], no MN to mild MN is indicated by a MIS < 9, moderate MN is indicated by a MIS of 9–18 and severe MN is indicated by a MIS > 18.

A pilot study was performed in 15 HDP to ensure all items were clear and easy to understand.

### Ethical approval

The institutional review boards (IRB) of the An-Najah National University and the local health authorities approved data collection and granted permission to access and use the patient’s clinical data.

### Statistical analysis

Statistical Package for the Social Sciences (SPSS) version 21 was used to enter and analyse the data. Data were expressed as frequencies (percentages) for categorical variables and as means ± SD or medians (lower–upper quartiles) for continuous variables. Nonparametric Mann–Whitney tests were used to compare two independent variables, and Kruskal–Wallis tests were used to compare multicategory variables (i.e. more than two independent variables) where appropriate. Kolmogorov–Smirnov tests were used to assess normality. The significance level was set at *P* < 0.05. Multiple linear regression models were used to identify the factors associated with MIS. Multiple linear regression model was built with selected MIS scores as the dependent variables. In addition, all univariate variables significant at *p* < 0.05 including age, living status, dialysis vintage, the number of chronic medications and the number of comorbid diseases were entered into a multiple linear regression model to adjust for the effects of confounding variables. The variance inflation factor (VIF) was used to detect the multicollinearity between independent variables in the regression model.

## Results

Of the 192 HDP approached, 18 refused to participate or failed to meet the inclusion criteria, thus the final HD sample consisted of 174 HDP (91 male). Patients were aged 57.7 ± 12.8 years, and the median dialysis vintage was 3 years (interquartile range 1–5 years). Most patients were town dwellers (44.8%), village dwellers (39.7%) or camp dwellers (15.5%). Regarding the patient’s educational level, approximately 6% did not have a formal education, approximately 33% finished their primary education and the remainder completed higher levels of education. Most patients (88.5%) did not earn an income (either unemployed or a housewife), and approximately 66% had a monthly income of < 2000 New Israeli Shekel (NIS). Most patients were married and lived with their families. A total of 59.2% of patients had been receiving dialysis for < 4 years, while the remaining 40.8% had been receiving HD for ≥4 years. Almost all patients (94.8%) received three sessions of HD per week, and 52.3% of these sessions persisted for < 4 h, while 47.7% persisted for ≥4 h. Only 9.8% of patients had previously received a kidney transplant. Regarding the patient’s BMI, 35.1% were within the normal range, 30.5% were overweight, 31.6% were obese and 2.9% were underweight. Most patients (> 90%) had at least one chronic comorbid disease (i.e. diabetes or hypertension) in addition to ESRD. A total of 60.9% of patients took four or more medications and 39.1% took less than four medications (Table 1).

**Table 1:** Sociodemographic and clinical characteristics of participants (*n* = 174)

Variable	Number of participants (%)
Age category (years)	
< 60	83 (47.7)
≥ 60	91 (52.3)
Gender	
Male	91 (52.3)
Female	83 (47.7)
BMI	
Underweight	5 (2.9)
Normal	61 (35.1)
Overweight	53 (30.5)
Obese	55 (31.6)
Education	
No formal education	11 (6.3)
Primary	63 (36.2)
Secondary	33 (19)
High	34 (19.5)
Graduate	33 (19)
Household income per month	
High (> 5000 NIS^a^)	6 (3.4)
Moderate (2000–4999 NIS)	52 (29.9)
Low (< 2000 NIS)	116 (66.7)
Residency	
Camps	27 (15.5)
Village	69 (39.7)
Town	78 (44.8)
Living status	
Alone	10 (5.7)
With family	164 (94.3)
Marital status	
Single, divorced or widowed	38 (21.8)
Married	136 (78.2)
Occupation	
Employed	20 (11.5)
Unemployed	134 (77)
Housewife	20 (11.5)
Dialysis vintage (years)	
< 4	103 (59.2)
≥ 4	71 (40.8)
Dialysis session per week	
≤ 2	5 (2.9)
3	165 (94.8)
≥ 4	4 (2.3)
Dialysis session duration (hours)	
< 4	91 (52.3)
≥ 4	83 (47.7)
Transplantation history	
Yes	17 (9.8)
No	157 (90.2)
Total number of chronic diseases	
< 3	118 (67.8)
≥ 3	56 (32.2)
Total number of chronic medications	
< 4	68 (39.1)
≥ 4	106 (60.9)

*BMI* body mass index, *NIS* New Israeli Shekel

^a^ 1 New Israeli Shekel = 0.29 US Dollar

To assess MN, measurements such as MIS, BMI, muscle mass, subcutaneous fat mass, plasma albumin and TIBC were considered. According to the previously mentioned measurements, 65% of patients were moderately malnourished, 34% were mildly malnourished and the remaining 1% were suffering from severe MN.

No significant association was found between a patient’s BMI and their nutritional status (*P* = 0.608). A significantly higher number of patients aged ≥60 years were malnourished compared to those aged < 60 years (*P* = 0.010). More patients who lived alone experienced MN compared to those who lived with their families (*P* = 0.032). MN was significantly higher among those who had received HD for ≥4 years (*P* = 0.008). HDP with increase in the number of comorbidities and the number of chronic medications taken had a significantly higher levels of MIS (*P* < 0.001 and *P* = 0.03, respectively) compared to those with fewer comorbidities and those taking fewer medications (Table 2).

**Table 2:** Relationship of malnutrition inflammation score (MIS)^a^ with sociodemographic and clinical characteristics in haemodialysis patients (*n* = 174)

Variable	Number of participants (%)	Median of MIS [Q1–Q3]	*P* value^b^
Age category (years)			**0.010** ^**c**^
< 60	83 (47.7)	9 [7–12]	
≥ 60	91 (52.3)	11 [8–14]	
Gender			0.639^c^
Male	91 (52.3)	10 [8–13]	
Female	83 (47.7)	10 [8–13]	
BMI			0.608^d^
Underweight	5 (2.9)	10 [9–14]	
Normal	61 (35.1)	10 [7–12]	
Overweight	53 (30.5)	11 [8–13]	
Obese	55 (31.6)	10 [8–13]	
Education			0.140^d^
No formal education	11 (6.3)	12 [8–14]	
Primary	63 (36.2)	10 [8–13]	
Secondary	33 (19)	11 [8–13]	
High	34 (19.5)	10 [8–12]	
Graduate	33 (19)	9 [5–11]	
Household income per month			0.464^d^
High (> 5000 NIS^e^)	6 (3.4)	9 [6–13]	
Moderate (2000–4999 NIS)	52 (29.9)	10 [7–12]	
Low (< 2000 NIS)	116 (66.7)	10 [8–13]	
Residency			0.623^d^
Camps	27 (15.5)	11 [8–13]	
Villages	69 (39.7)	10 [8–13]	
Towns	78 (44.8)	10 [8–12]	
Living status			**0.032** ^**c**^
Alone	10 (5.7)	15 [8–16]	
With family	164 (94.3)	10 [8–13]	
Marital status			0.364^c^
Single, divorced or widowed	38 (21.8)	11 [8–13]	
Married	136 (78.2)	10 [8–13]	
Occupation			0.211^d^
Employed	20 (11.5)	10 [8–13]	
Unemployed	134 (77)	9 [5–12]	
Housewife	20 (11.5)	10 [8–13]	
Dialysis vintage (years)			**0.008** ^**c**^
< 4	103 (59.2)	9 [7–12]	
≥ 4	71 (40.8)	11 [9–14]	
Dialysis sessions per week			0.173^d^
≤ 2	5 (2.9)	8 [5.5–9]	
3	165 (94.8)	10 [8–13]	
≥ 4	4 (2.3)	11 [6–13]	
Dialysis session duration (hours)			0.178 ^c^
< 4	91 (52.3)	10 [8–13]	
≥ 4	83 (47.7)	10 [8–12]	
Transplantation history			0.384^c^
Yes	17 (9.8)	9 [8–12]	
No	157 (90.2)	10 [8–13]	
Total number of chronic diseases			**0.001** ^**c**^
< 3	118 (67.8)	7 [7–12]	
≥ 3	56 (32.2)	11 [9–14]	
Total number of chronic medications			**0.030** ^**c**^
< 4	68 (39.1)	9 [7–12]	
≥ 4	106 (60.9)	11 [8–13]	

*BMI* body mass index, *NIS* New Israeli Shekel; Q1–Q3, first quartile–third quartile

^a^The malnutrition-inflammation scale score ranges from 0 to 30, with high scores reflecting a more severe degree of malnutrition and inflammation status

^b^*P* values in bold are below the significance level of 0.05

^c^Statistical significance values calculated using the Mann–Whitney U test

^d^Statistical significance values calculated using the Kruskal–Wallis test

^e^1 Israeli New Shekel = 0.29 US Dollar

In a multivariable linear regression model, the MIS was independently associated with age, living status, dialysis vintage, the number of chronic medications and the number of comorbid diseases (adjusted R2, 0.215; *P* < 0.010); (Table 3). More specifically, patients with HD, especially those who were elderly (unstandardized coefficient β, 1.728; 95% CI, 0.700 to 2.756; *P* = 0.001), those with multiple comorbid diseases (unstandardized coefficient β, 1.673; 95% CI, 0.556 to 2.789; *P* = 0.004); those taking multiple chronic medications (unstandardized coefficient β, 1.259; 95% CI, 0.197 to 2.321; *P* = 0.020), or those with a long dialysis vintage (unstandardized coefficient β, 1.449; 95% CI, 0.410 to 2.487; *P* = 0.007), were positively associated with the MIS score in a multivariable linear regression model. Furthermore, HDP living with their family were negatively associated with the MIS score (unstandardized coefficient β, − 2.545; 95% CI, − 4.738 to − 0.352; *P* = 0.023); (Table 3). No problem multicollinearity between the independent variables was observed.

**Table 3:** Multivariable linear regression analysis showing independent variables associated with the MIS ^a^ score in haemodialysis patients

Variables ^b, c^	Unstandardized Coefficients ^d^		Standardized Coefficients ^e^	t	*P* value ^f^	95% confidence interval for B
	B	Std. Error	Beta			
Age category (years)						
< 60	Ref. 1.728	0.521	0.229	3.318	**0.001**	0.700 to 2.756
≥ 60						
Living status						
Alone	Ref. −2.545	1.111	−0.157	−2.291	**0.023**	−4.738 to −0.352
With family						
Total number of chronic diseases						
< 3	Ref. 1.673	0.566	0.207	2.958	**0.004**	0.556 to 2.789
≥ 3						
Total number of chronic medications						
< 4	Ref. 1.259	0.538	0.163	2.341	**0.020**	0.197 to 2.321
≥ 4						
Dialysis vintage (years)						
< 4	Ref. 1.449	0.526	0.189	2.754	**0.007**	0.410 to 2.487
≥ 4						

^a^The malnutrition-inflammation scale (MIS) is a 10-component scale with a higher score reflecting a more severe degree of malnutrition and inflammation status

^b^Univariate variables with *P* values < 0.05 were entered into the multiple linear regression

^c^Nominal variables were entered into analysis using dummy coding

^d^An unstandardized coefficient (B) represents the amount of change in a dependent variable (i.e. MIS score) due to a change of 1 unit for each of independent variable

^e^A standardized coefficient (Beta) compares the power of the effect of each individual independent variable to the dependent variable. The independent variable with a higher absolute value of beta coefficient has a stronger effect

^f^*P* values are bold where they are less than the significance level of 0.05

## Discussion

In Palestine, studies on the nutritional status of HDP have not yet been published. Therefore this study is the first to explore the prevalence of MN among HDP in Palestine using an inexpensive nutritional assessment tool. The MIS score consisted of four main elements: the patient’s related medical history, their BMI, a physical examination and laboratory parameters [10].

Our results indicated that most (65%) patients were moderately malnourished. This is higher than reported results in other Middle Eastern countries. Naini et al., performed a case–control study in 2011–12 at the Isfahan University of Medical Sciences, Iran, using the same score cut-off point and found that 72.7% of patients were mildly malnourished while 25.5% were moderately malnourished [29].

In the current study, MN was significantly affected by the patient’s age, whether they lived alone or with family, their dialysis vintage, the total number of chronic comorbid diseases, and the total number of chronic medications used.

In some aspects, our results are in agreement with other related published studies. For example, our study indicated that MN was significantly higher among the group aged > 60 years. Similarly, Alharbi and Enrione performed a similar study to investigate the prevalence of MN among HDP at the Jeddah Kidney Center, Jeddah, Saudi Arabia, and reported that older HDP (≥55 years) tended to be malnourished [30]. Oliveira et al. also conducted a study to evaluate the nutritional status in HDP and reported that there was a significantly increased prevalence of MN among the elderly compared to those younger than 60 years [31]. The findings that MN was more prevalent in the elderly could be explained by the hardship faced by those patients during this period of life, such as reduced mobility, reduced cognition, loss of appetite, decreased taste, difficulty buying food and preparing meals, poor dental hygiene, an increased prevalence of chronic or acute diseases and diminished food intake [31].

The most obvious finding to emerge from our analysis is that patients who underwent HD for > 4 years were more likely to develop MN. Corroborating one study by Chumlea [32], which set out to determine the prevalence of MN and factors associated with HDP, Freitas et al. [33] found a strong correlation between long HD duration and poor nutritional status. In addition, HD is a high catabolic process that promotes a significant loss of essential nutrients, such as amino acids, vitamins, proteins and glucose [34]. The nutritional status may deteriorate over time if these nutrients are not sufficiently replenished. Bohé and Rennie provided a significant analysis and discussion on the subject and reported that patients receiving dialysis three times a week lost 2 kg of lean body mass in a year [35].

It is interesting to note that that patients living alone were more malnourished than patients living with their family in our study. Living within a family framework is one of the factors that may improve home care. CKD worsens the functional activity and compromises the autonomy of individuals, thus making them completely or partially dependent on another person, and this occurs more frequently in the elderly [26, 36, 37]. Patients taking more chronic medications were also more susceptible to MN, which may be due to the fact that increasing numbers of medications result from increased numbers of comorbidities [38]. Another important finding in our study that comorbidities was significant predictors of malnutrition in HDP. Similar to our study, Jahromi et al. showed that, using Dialysis Malnutrition Score, poor protein and energy intake, comorbidities and inflammation were the predictors of MN in descending order of importance [39]. Morais et al. performed a prospective study to investigate the association between nutritional status and food intake in HDP and the authors reported that comorbidities had a significant correlation with subjective global assessment, which was similar to the findings reported here [40]. This may contribute to a reduced nutritional intake or the promotion of a higher catabolism state.

The current study found that measured variables including gender, BMI, education, income, residency, marital status, occupation, dialysis sessions per week, and transplantation history were not significantly associated with MN status in HDP. However, Ekramzade et al. reported that HDP with MN had a lower BMI than well-nourished patients [41]. The prevalence of MN was not related to gender in a cross-sectional study, in a study by Santos et al. that investigated serum albumin as an index of nutritional status in HDP [42], in an observational study conducted by Elliot and Robb [43] or in a study investigating the nutritional status of patients undergoing HD by Oliveira et al. [31]. Oliveira et al. performed a detailed assessment of the nutritional status in HDP and showed that MN was correlated with monthly personal income [31], which was in opposition to the data reported here. Contrary to this study, the cross-sectional study by Oliveira et al. also reported that MN was more prevalent among illiterate patients that those with a higher education status [31].

## Strengths and limitations

The prevalence of MN in HDP has not yet been documented in Palestine. This study was the first to detect MN among HDP in Palestine using an inexpensive nutritional assessment known as the MIS score. However, this study has some limitations; for example, the fact that a cross-sectional design was used limits the ability to make causal inferences between study variables. Moreover, as participants were only recruited from one HD centre by convenience sampling, the findings cannot be generalized to all patients with HD in Palestine. Selection bias also cannot be entirely dismissed as participation in the study was voluntary. Another limitation was that the MIS is considered as a subjective tool for the evaluation of nutritional status, therefore it mainly relies on the assessment and clinical judgment of each examiner. Lastly, there were some clinical factors that might affect patients’ nutritional status and potential weaknesses of the study that we didn’t include in our study, such as the presence of residual renal function (albeit long dialysis vintage is the surrogate potentially for such) or total weekly dialysis time.

## Conclusions

The MIS score results indicated that MN was prevalent among patients undergoing HD. These results show some correlations between the nutritional status and patient characteristics (i.e. clinical and sociodemographic factors). Therefore these findings should increase the awareness of healthcare providers for interventions to enhance the nutritional status for HDP, especially those who are elderly, have multiple comorbid diseases, take multiple chronic medications, have a long dialysis vintage or live alone. Consequently, efficient screening in HDP for risk factors of MN and simultaneously performing a nutritional evaluation and assessment whenever possible should facilitate early dietary intervention to avoid further deterioration and nutritional depletion. Further interventional studies should be conducted in more than one centre and with a larger sample size to repeat these results so that they are proven with greater certainty.

## Additional file

Additional file 1: Study questionnaire. This is the final version of the English version that was used to obtain data which will help to assess the nutritional status among haemodialysis patients and to establish the factors associated with malnutrition in these patients. (DOCX 60 kb)

## Abbreviations

BMI: Body mass index
CKD: Chronic kidney disease
CRP: C-reactive protein
ESRD: End-stage renal disease
HD: Haemodialysis
HDP: Haemodialysis patients
IRB: Institutional review boards
MIS: Malnutrition-inflammation score
MN: Malnutrition
NIS: New Israeli Shekel
PEW: Protein-energy wasting
SPSS: Statistical package for social sciences
TIBC: Total iron binding capacity
VIF: Variance inflation factor
