# Supplementary material for: Assessment of nutritional status in the maintenance of haemodialysis patients: a cross-sectional study from Palestine
Source: BMC Nephrol. 2019 Mar 15;20:92. doi: 10.1186/s12882-019-1288-z (PMC6420767; doi:10.1186/s12882-019-1288-z)
Supplement: Supplementary file 1 — Study questionnaire. This is the final version of the English version that was used to obtain data which will help to assess the nutritional status among haemodialysis patients and to establish the factors associated with malnutrition in these patients. (DOCX 60 kb) [file 12882_2019_1288_MOESM1_ESM.docx]

**Additional file 1: Study questionnaires. This is the final version of the English version that was used to obtain data which will help to assess the nutritional status among haemodialysis patients and to establish the factors associated with malnutrition in these patients.**

**English version**

**Section one:**

1-**age:** ………………

2- **Sex:**  Male  Female

3- Height: …...... 4 – Weight: ..........

5- **Residency:**  Camp  Village  City

6- **Your current residence:**  I live alone  I live with my family  Other

7- **What is your level of education:**  Not educated  primary  Secondary

 College (diploma)  Graduates (BA)  Postgraduate studies

8- **Marital status:**  Single  Married  Divorced  Widowed

9- **What is your business:**  I do not work  Employee  Non-employee Housewife

10- **How much is the average monthly income of a family:**  Less than 2000 NIS  2000-5000 5000-10000  Over 10000 NIS

**Section two:**

11- Dialysis vintage years ……………………

12- **How many times you go for dialysis weekly**

two times or less  three times

 four times  five times  more than five times, ...........................

13- How many hours each session takes …………………

14- Have you done kidney transplant before  Yes  No

15- **Do have any other disease :**

 Diabetes  Hypertension  Arthritis  Systemic Lupus Erythematous

 Angina  Stroke Heart failure  Chronic lung disease

Other diseases……………………………………………….

16- **What medications do you take**

1…………….…..…..2-…………………. 3- .......................

4-……………………5-………………..…6-..........................

Others…………………………………………..

17- **Do you take your medication by yourself?** Yes No

18- **Are you a smoker?** Yes No  ex-smoker

19- If your answer is yes , how many cigarettes per day? ………..

**Section three:**

| **MALNUTRITION INFLAMMATION SCORE (M.I.S)** | | | |
| --- | --- | --- | --- |
| 1. **Patient's related medical history:** 2. **Change in end dialysis dry weight (overall change in past 3-6 months):** | | | |
| 3  weight loss > 5% | 2  Weight loss more than 1 kg but < 5% | 1  Minor weight loss (≥ 0.5 kg but < 1 kg) | 0  No decrease in dry weight or weight loss < 9.5 kg |
| 1. **Dietary Intake:** | | | |
| 3  Hypo-caloric liquid to starvation | 2  Moderate overall decrease to full liquid diet | 1  Somewhat sub-optimal solid diet intake | 0  Good appetite and and no deterioration of the dietary intake pattern |
| 1. **Gastrointestinal (GI) symptoms:** | | | |
| 3  Frequent diarrhea or vomiting or severe anorexia | 2  Occasional vomiting or moderate GI symptoms | 1  Mild symptoms, poor appetite or nauseated occasionally | 0  No symptoms with good appetite |
| 1. **Functional capacity (nutritionally related functional impairment)::** | | | |
| 3  Bed/chair-ridden,  or little to no physical activity | 2  Difficulty with otherwise independent activities (e.g. going to bathroom) | 1  Occasional difficulty with baseline ambulation,  or feeling tired frequently | 0  Normal to improved functional capacity, feeling fine |
| 1. **Co-morbidity, including number of years on dialysis:** | | | |
| 3  Any severe, multiple co-morbidity (2 or more MCC*) | 2  Dialyzed > 4 years, or moderate co-morbidity (including one MCC*) | 1  Dialyzed for 1-4 years, or mild co-morbidity (excluding MCC*) | 0  On dialysis < 1 year and healthy otherwise |
| 1. **Physical Exam (according to SGA Crieteria):** 2. **Decreased fat stores or loss of subcutaneous fat (below eyes, triceps, biceps, chest):** | | | |
| 3  Severe | 2  Moderate | 1  Mild | 0  Normal (no change) |
| 1. **Signs of muscle wasting (temple, clavicle, scapula, ribs, quadriceps, knee, interosseous):** | | | |
| 3  Severe | 2  Moderate | 1  Mild | 0  Normal (no change) |
| 1. **Body Mass Index:** 2. **Body mass index** | | | |
| 3  BMI < 16 kg/m^2^ | 2  BMI 16 - 17.99 kg/m^2^ | 1  BMI 18 - 19.99 kg/m^2^ | 0  BMI ≥ 20 kg/m^2^ |
| 1. **Laboratory Parameters:** 2. **Serum albumin** | | | |
| 3  Albumin < 3.0 g/dL | 2  Albumin 3.0 - 3.4 g/dL | 1  Albumin 3.5 - 3.9 g/dL | 0  Albumin ≥ 4.0 g/dL |
| **10. Serum TIBC (Total Iron Binding Capacity) OR serum transferrin** | | | |
| 3  TIBC < 150 mg/dL Transferrin < 150 mg/d | 2  TIBC 150 -199 mg/dL Transferrin 150 -169 mg/dL | 1  TIBC 200 -249 mg/dL Transferrin 170 -200 mg/dL | 0  TIBC ≥ 250 mg/dL Transferrin > 200 mg/dL |
| ***MCC (Major Comorbid Conditions): include CHF class III or IV, full blown AIDS severe CAD, moderate to severe COPD, major neurologic sequelae, and metastatic malignancies or s/p recent chemotherapy.** | | | |

**Adapted from** [**http://www.touchcalc.com/calculators/mis**](http://www.touchcalc.com/calculators/mis) **based on** Kalantar-Zadeh et al (2001)

Kalantar-Zadeh K, Kopple JD, Block G, Humphreys MH: **A malnutrition-inflammation score is correlated with morbidity and mortality in maintenance hemodialysis patients**. *Am J Kidney Dis* 2001, **38**(6):1251-1263.
